# Considerations on the Castrop formula for calculation of intraocular lens power

**DOI:** 10.1371/journal.pone.0252102

**Published:** 2021-06-02

**Authors:** Achim Langenbucher, Nóra Szentmáry, Alan Cayless, Johannes Weisensee, Ekkehard Fabian, Jascha Wendelstein, Peter Hoffmann

**Affiliations:** 1 Department of Experimental Ophthalmology, Saarland University, Homburg, Saar, Germany; 2 Dr. Rolf M. Schwiete Center for Limbal Stem Cell and Aniridia Research, Saarland University, Homburg, Saar, Germany; 3 Department of Ophthalmology, Semmelweis-University, Budapest, Hungary; 4 School of Physical Sciences, The Open University, Milton Keynes, United Kingdom; 5 Augencentrum Rosenheim, Rosenheim, Deutschland; 6 Department of Ophthalmology, Johannes Kepler University Linz, Linz, Austria; 7 Augen- und Laserklinik Castrop-Rauxel, Castrop-Rauxel, Germany; Edith Wolfson Medical Center, ISRAEL

## Abstract

**Background:**

To explain the concept of the Castrop lens power calculation formula and show the application and results from a large dataset compared to classical formulae.

**Methods:**

The Castrop vergence formula is based on a pseudophakic model eye with 4 refractive surfaces. This was compared against the SRKT, Hoffer-Q, Holladay1, simplified Haigis with 1 optimized constant and Haigis formula with 3 optimized constants. A large dataset of preoperative biometric values, lens power data and postoperative refraction data was split into training and test sets. The training data were used for formula constant optimization, and the test data for cross-validation. Constant optimization was performed for all formulae using nonlinear optimization, minimising root mean squared prediction error.

**Results:**

The constants for all formulae were derived with the Levenberg-Marquardt algorithm. Applying these constants to the test data, the Castrop formula showed a slightly better performance compared to the classical formulae in terms of prediction error and absolute prediction error. Using the Castrop formula, the standard deviation of the prediction error was lowest at 0.45 dpt, and 95% of all eyes in the test data were within the limit of 0.9 dpt of prediction error.

**Conclusion:**

The calculation concept of the Castrop formula and one potential option for optimization of the 3 Castrop formula constants (C, H, and R) are presented. In a large dataset of 1452 data points the performance of the Castrop formula was slightly superior to the respective results of the classical formulae such as SRKT, Hoffer-Q, Holladay1 or Haigis.

## Introduction

Since the introduction of biometry instruments for measuring axial eye length and publication of the first formulae by Fyodorov in 1967 [1] and by Gernet in 1970 [2], many calculation schemes have been proposed for predicting the power of an artificial lens in cataract surgery. The simplest are the SRK and SRK2 formulae [3], which use a linear model to derive the emmetropic lens power from the axial length measurement, the ‘keratometry reading’ and the A constant used as an intercept. In the SRK2, the A constant is modified by an offset depending on the axial length. The classical theoretical-optical formulae in use today are the SRKT [4, 5], Hoffer-Q [6–8], Holladay1 [9], and the Haigis formula [3]. The latter is sometimes used in a simplified form with standard values for constants a1 = 0.4 and a2 = 0.1 and optimization of a0 only, or in a version with triple constant optimization a0/a1/a2 which appears to yield superior results in long and short eyes.

In addition to these formulae, published in the 1980s and 1990s, many other calculation concepts have been proposed. Most of these are again based on calculations with paraxial simplifications, and most of them have never been published such as the Holladay2 or the Barrett Universal II formula. In addition, several formulae have been developed to consider the situation after keratorefractive surgery (e.g. the Barrett TrueK, Haigis-L, Masket, Shammas formula or others), where most of the classical formulae fail. Some other calculation strategies are based on full aperture raytracing without simplifications to linear Gaussian optics (e.g. Okulix or PhakoOptics) or on artificial intelligence (e.g. Hill RBF calculator or PEARL formula), where a large dataset replaces an anatomical or optical model [10]. All of these concepts claim to yield superior results to other formulae.

In general, it is difficult to check the performance of different formulae, as it is mostly dependent on the composition of the dataset, and there are no common rules on how to evaluate the performance. Therefore, some formulae are superior in long or short eyes [11, 12], or in eyes with a steeper or flatter cornea, or selected combinations of axial length and corneal curvature. Additionally, the performance of a formula mostly depends on the dataset used for constant optimization as well as the technique applied for optimizing the constants, and it can also depend on the biometer settings, quality of the clinical measurement data (e.g. refraction and lane distance), and the labeling tolerance of the lens [13–15]. As target criteria for evaluating the performance of a specific formula we use the prediction error (PE) in terms of achieved refraction after cataract surgery (SE) minus the formula prediction (predSE), the absolute value of the prediction error (absPE), or the portion of cases within specific refraction limits (e.g. ≤±0.25, ≤±0.5, or ≤±1.0 dpt) [16]. With the PE we mostly obtain the systematic deviation of the achieved refraction from the formula prediction, e.g. if a study population systematically results in hyperopia or myopia. In contrast, the absPE mostly refers to the variation or scatter of the results, but a proper interpretation of absPE as variation of the results presupposes that the mean or median PE is close to zero [14]. The PE is typically a two-sided distribution, and if close to a normal distribution would be fully characterized by the mean and standard deviation. In contrast, the absPE is a strictly one-sided distribution, where the mean and standard deviation do not have a direct meaning, and in this case the median or confidence levels should be used for characterization [16].

Measurements in previous generations of biometers were restricted to axial length, corneal front surface curvature, anterior chamber depth, and optionally the horizontal corneal diameter and the formulae of that era focused on the parameters available at that time [3]. Most modern biometers provide additional information about corneal thickness, corneal back surface curvature, aqueous depth, central thickness of the crystalline lens, and vitreous depth [17]. Furthermore, some biometers analyze corneal topography or even tomography data at many points, providing information on the asphericity in addition to the curvature of the front or front and back surfaces. In the future, with consistent tomographic data available from most biometers, there might be a quantum leap towards full aperture raytracing in routine intraocular lens power calculations.

The purpose of the present study is:

- to introduce and explain the principle and calculation scheme of the Castrop formula; a paraxial vergence formula for intraocular lens power calculation,
- to assess the performance of the Castrop formula with an explorative data analysis applied to a large dataset of a cataract population with preoperative biometric data, the power of the implanted lens, and postoperative refraction data using cross validation techniques, and
- to compare the results of the Castrop formula with the respective results of classical formulae such as SRKT, Hoffer-Q, Holladay1, and the Haigis formula.

## Materials and methods

### The Castrop IOL calculation concept

The Castrop formula is a paraxial vergence formula based on a pseudophakic model eye with 4 refractive surfaces and 3 formula constants (C, H, and R): a refractive correction at the spectacle plane, a thick cornea with front and back surfaces, and a thin intraocular lens (IOL). Castrop-Rauxel is a city in the middle west of Germany in the federal state of North Rhine Westphalia. The respective refractive powers of these elements are: predSE for the predicted spherical equivalent refraction, P_CA_ = (n_C_-1)/R_CA_ and P_CP_ = (n-n_C_)/R_CP_ for the corneal front and back surface power, and P_IOL_ for the lens implant. R_CA_ and R_CP_ refer to the corneal front and back surface radii of curvature, the vertex distance d_VD_ refers to the interspace between the spectacle plane and the corneal front vertex plane, d_C_ to the central corneal thickness, d_AQD_ to the aqueous depth, and d_V_ to the vitreous, respectively. For the refractive index we used n_C_ = 1.376 (cornea), and n = 1.336 for aqueous humour and vitreous humour derived from a common schematic model eye, respectively [17].

To account for the effect of refraction measurement lane distance or systematic error in refraction measurement, an offset of R was superimposed on the refraction term ta achieve predSE. The IOL is assumed to be located within the envelope of the crystalline lens, similar to the recommendation of the lens haptic plane concept of Norrby [18] or the Olsen formula [19, 20]: The effective lens position ELP, defined as the distance of the corneal front vertex plane to the plane of the IOL, is derived from the preoperative phakic anterior chamber depth (ACD), the central thickness of the crystalline lens (LT), and an axial offset (H) as:

$$ELP=d_{C}+d_{AQD}=ACD+C∙LT+H,$$

where C refers to the (anterior) portion of the thickness of the crystalline lens LT to the lens equator. C is typically in a range of 0.36 to 0.44.

With AL as a measure for the axial length of the eye, the vergence formula for the IOL power reads:

$$P_{IOL}=\frac{n-1}{AL-ACD-C∙LT-H}-\frac{1}{\frac{1}{\frac{1}{\frac{1}{\frac{1}{\frac{\text{preSE}-R}{1-(\text{predSE}-R)∙d_{VD}}}\;+\;\frac{n_{C}-1}{R_{CA}}}\;-\;\frac{d_{C}}{n_{C}}}\;+\;\frac{{n-n}_{C}}{R_{CP}}}-\frac{ACD-d_{C}+C∙LT+H}{n}}$$

In total the Castrop formula is characterized with 3 formula constants, C, H, and R. Solving the equation for the predicted refraction predSE yields:

$$\text{predSE}=\frac{1}{\frac{1}{\frac{1}{\frac{1}{\frac{1}{\frac{1}{\frac{n-1}{AL-ACD-C∙LT-H}\;-\;P_{IOL}}\;+\;\frac{ACD-d_{C}+C∙LT+H}{n}}\;-\;\frac{{n-n}_{C}}{R_{CP}}}\;+\;\frac{d_{C}}{n_{C}}}\;-\;\frac{n_{C}-1}{R_{CA}}}+d_{VD}}+R$$

In cases where measurements of R_CP_ or d_C_ are not available or not reliable, the respective data can be derived from a schematic model eye [17] maintaining a fixed ratio of corneal front to back surface curvature:

$$\begin{array}{ccc} R_{CP} & = & R_{CA}∙\frac{6.4}{7.77}\\ d_{C} & = & 500\;\mu \text{m} \end{array}$$

### Formula constant optimization

There are different potential strategies for optimizing C, H and R. The constants could be optimized in turn (e.g. first the C, then the H, and then R), or *en bloc* using an enhanced optimization strategy as commonly used in engineering. As the effects of the 3 constants are not independent (similar to the Haigis 3 constant optimization) we decided in this study to use a nonlinear iterative optimization strategy. As target measures for optimization we considered the mean absolute prediction error (mean absPE) and the root mean squared PE (rmsPE). The Levenberg-Marquardt algorithm [21, 22] was initialized with the formula constant triplet C/H/R = 0.4/0/0 with boundary conditions 0.35 to 0.45/-0.8 to 0.8, and -0.8 to 0.8. We limited iterations to a maximum of 100, with a damping of 1e-2, a termination step size of 1e-14 (optimization ends when last step is smaller than 1e-14), and a function tolerance of 1e-16 (optimization ends if rmsPE improvement is smaller than 1e-16).

### Measurement data

In this retrospective study we analysed a dataset with 1601 clinical data points from a cataract population from Augencentrum Rosenheim which was transferred to us (802 left eyes and 799 right eyes; 961 female and 640 male). Mean age was 74.2±8.1 years, median: 75 years, range: 47 to 97 years). According to the statement from the ‘Bayerische Landesärztekammer’, an ethics approval or patient informed consent was not required for this study. This study is a retrospective evaluation of data which were collected during routine examinations. No extra examinations or measurements were performed.

The data were transferred to us in an anonymized fashion, which precludes back-tracing of the patient. The anonymized data contained preoperative biometric data derived with the IOLMaster 700 (Carl-Zeiss-Meditec, Jena, Germany) including axial length (AL), central corneal thickness d_C_, external phakic anterior chamber depth ACD measured from the corneal front apex to the anterior apex of the crystalline lens, lens thickness LT, corneal front surface radius measured in the flat (R1) and in the steep meridian (R2). In all cases a Sensar 1 piece intraocular lens (Johnson & Johnson, Brunswick, USA) was inserted. In addition to the refractive power of the inserted lens (P_IOL_), the postoperative refraction (sphere and cylinder) 6 to 8 weeks after cataract surgery was recorded. We restricted the dataset to postoperative data with a visual acuity of 0.6 or higher to ensure that the postoperative refraction was reliable. It is shown in the literature that with a low visual acuity e.g. due to retinal pathologies refractometry might be unreliable [16]. From the original dataset, a subset of N = 1452 complete data points were used for formula constant optimization. The spherical equivalent of postoperative refraction (SE) was calculated as sphere + ½·cylinder, and the mean corneal front surface radius was calculated as R_CA_ = ½ (R1+R2). The corneal back surface curvature was calculated from the corneal front surface curvature as shown above using a fixed ratio of front to back surface radius. Axial length was pre-processed using the Cooke correction [23, 24]. The descriptive data on pre-cataract biometry, P_IOL_ and postoperative refraction are summarized in Table 1.

**Table 1:** Descriptive statistics of the entire dataset with mean, standard deviation (SD), median, minimum and maximum, 5%, and 95% quantiles (90% confidence intervals).

N = 1452	AL in mm	ACD in mm	LT in mm	R_CA_ in mm	P_IOL_ in D	SE in D
Mean	23.497	3.099	4.608	7.668	21.519	-0.509
SD	1.082	0.389	0.422	0.271	2.849	0.859
Median	23.419	3.101	4.606	7.657	21.5	-0.375
Minimum	20.635	2.000	3.345	6.671	8.0	-4.0
Maximum	29.041	4.354	5.794	8.676	30.0	2.0
Quantile 5%	22.016	2.461	3.912	7.251	16.5	-2.5
Quantile 95%	25.529	3.753	5.311	8.134	26.0	0.5

AL refers to the axial length after Cooke correction (Cooke & Cooke 2019a; Cooke & Cooke 2019b), ACD to the external phakic anterior chamber depth measured from the corneal front apex to the front apex of the crystalline lens, LT to the central thickness of the crystalline lens, R_CA_ to the average corneal front surface radius, IOLP to the refractive power of the implanted lens, and SE to the spherical equivalent of postoperative refraction.

### Calculation strategy

The anonymized Excel data (.xlsx-format) were imported into MATLAB (Matlab version 2019b, The Math Works, Natick, USA) for further processing. For the Castrop formula we implemented the calculation strategy described above. To compare the formula performance to classical formulae we also implemented the SRKT formula [4, 5], the Hoffer-Q formula [6–8], the Holladay1 formula [9], the simplified Haigis with one formula constant a0 and standard values for a1 = 0.4 and a2 = 0.1, and the Haigis formula [3] with optimized constant triplet a0/a1/a2 [13]. For all calculations, the deviation of the achieved postoperative refraction SE from the formula predicted refraction predSE was quoted as the prediction error PE. The absPE refers to the absolute value of PE [16].

For cross validation, the N = 1452 measurements were split randomly into a training set (70%, 1017 cases) and a test set (30%, 435 cases) [25]. For the training set, the constants for the Castrop formula (C, H, R), the SRKT (A), the Hoffer-q (pACD), the Holladay1 (SF), the simplified Haigis (a0) and Haigis formula with triplet optimization (a0/a1/a2) were optimized in terms of minimizing the rmsPE using the nonlinear Levenberg-Marquardt algorithm [21, 22].

In the next step we calculated the mean and median absolute prediction error, and the root mean squared prediction error for all lens calculation formulae based on the optimized formula constants using the test dataset. Then for all formulae we derived the fraction of cases with absPE ≤ 0.25 dpt, ≤ 0.5 dpt, ≤ 1.0 dpt, and ≤ 2.0 dpt.

As an overall metric for formula performance we calculated the formula performance index FPI as described by Hoffer & Savini [16]. For estimation of the slope of the correlation of prediction error PE vs. axial length AL as one component of this index, we used a robust estimator with Studentized residuals.

The Wilcoxon signed rank test was used to compare the absPE of the Castrop formula to the absPE of other formulae. A p-value of <0.05 was considered statistically significant.

## Results

Based on the training set (N = 1017 measurements), the optimization of the formula constants for a minimal root mean squared prediction error rmsPE yielded the following values: SRKT: A = 119.011, Foffer-Q: pACD = 5.606, Holladay1: SF = 1.824, simplified Haigis with a1 = 0.4 and a2 = 0.1: a0 = 1.446, Haigis: a0/a1/a2 = 1.565/0.443/0.089, and Castrop: C = 0.424, H = -0.312, R = 0.077. For the Castrop formula, 26 iterations of the Levenberg-Marquardt algorithm were necessary to reach the termination criteria. The target criterion for optimization (sum of the squared prediction errors) was reduced during iteration from 265.1 dpt^2^ (initialization) to 201.4^2^ (optimized formula constants for the Castrop formula), which refers to a mean squared prediction error in the training set of 201.4/1017 = 0.198 dpt^2^, or a rmsPE of 0.445 dpt.

Table 2 summarizes the descriptive statistics for the prediction error PE for all formulae. The prediction error is derived using the constants optimized on the training data and cross-validated on the N = 435 test data. PE is listed in terms of mean, standard deviation, median, range, and 5% / 95% quantiles.

**Table 2:** Prediction error (×100) as the difference between achieved spherical equivalent and formula predicted spherical equivalent for the formulae under test.

N = 435 test data	Prediction error PE in dpt ×100					
	SRKT	Hoffer-Q	Holladay1	Simplified Haigis	Haigis	Castrop
Mean	1.86	2.1	1.4	1.1	1.2	0.6
Standard deviation	51.5	54.6	52.9	49.1	48.8	45.7
Median	3.2	5.2	2.7	2.2	0.3	1.4
Minimum	-203.4	-225.9	-213.4	-203.9	-201.4	-189.9
Maximum	244.4	214.0	275.6	202.6	202.3	210.6
5% quantile	-88.2	-88.0	-88.3	-78.8	-76.7	-71.1
95% quantile	73.2	83.0	77.3	75.2	75.1	67.4

The formula constants were optimized on the training dataset and cross validated on the test set.

**Fig 1:**
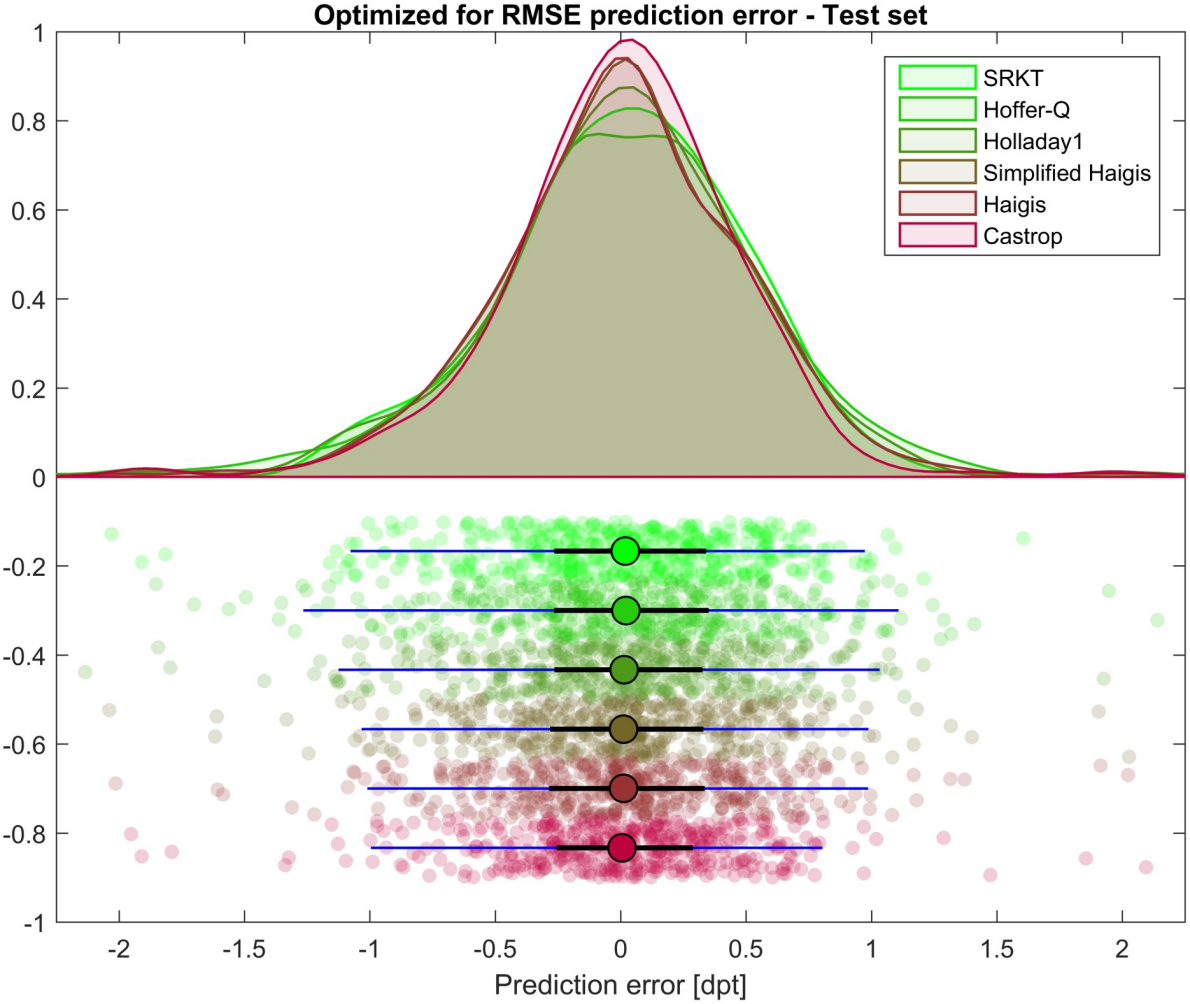
Prediction error PE as the deviation of achieved spherical equivalent from the formula predicted refraction. Formula constants have been optimized on the training dataset (N = 1017) and cross-validated on the test set (N = 435). The upper part of the plot shows the approximated kernel density distribution for the 6 formulae under test, and the lower part the scatter of the PE together with the median (circle), the quartiles (black line), and the 90% confidence interval (5% and 95% quantile, blue line). We see that after optimization of the formula constants on the training data none of the formulae shows a systematic offset in the prediction error. The blue lines in the lower plot representing the 90% confidence interval show that the variation on the test data is largest with the Hoffer-Q formula and lowest with the Castrop formula.

Fig 1 displays the scatter of the formula prediction error PE for the test data for all 6 formulae under test, together with the approximated kernel distribution, the median, the quartiles and the 90% confidence interval.

Table 3 summarizes the descriptive statistics for the absolute value of the prediction error absPE for all formulae. The absolute value of the prediction error is calculated using the constants optimized on the training data and cross-validated on the N = 435 test data. absPE is listed in terms of mean, standard deviation, median, range, and 5% / 95% quantiles.

**Table 3:** Absolute value of the prediction error (×100) as the difference between achieved spherical equivalent and formula predicted spherical equivalent for the formulae under test.

N = 435 test data	Absolute value of the prediction error absPE in dpt ×100					
	SRKT	Hoffer-Q	Holladay1	Simplified Haigis	Haigis	Castrop
Mean	38.8	41.7	39.0	36.8	36.7	34.0
Standard deviation	33.9	35.4	35.6	32.5	32.2	30.5
Median	30.5	30.8	30.6	30.2	29.9	27.2
Minimum	0.1	0.2	0.0	0.0	0.1	0.1
Maximum	244.7	225.9	275.6	203.9	202.3	210.6
5% quantile	2.4	5.0	2.8	2.3	2.0	2.4
95% quantile	99.2	110.5	103.6	97.0	94.7	89.7

The formula constants were optimized on the training dataset and cross validated on the test set.

**Fig 2:**
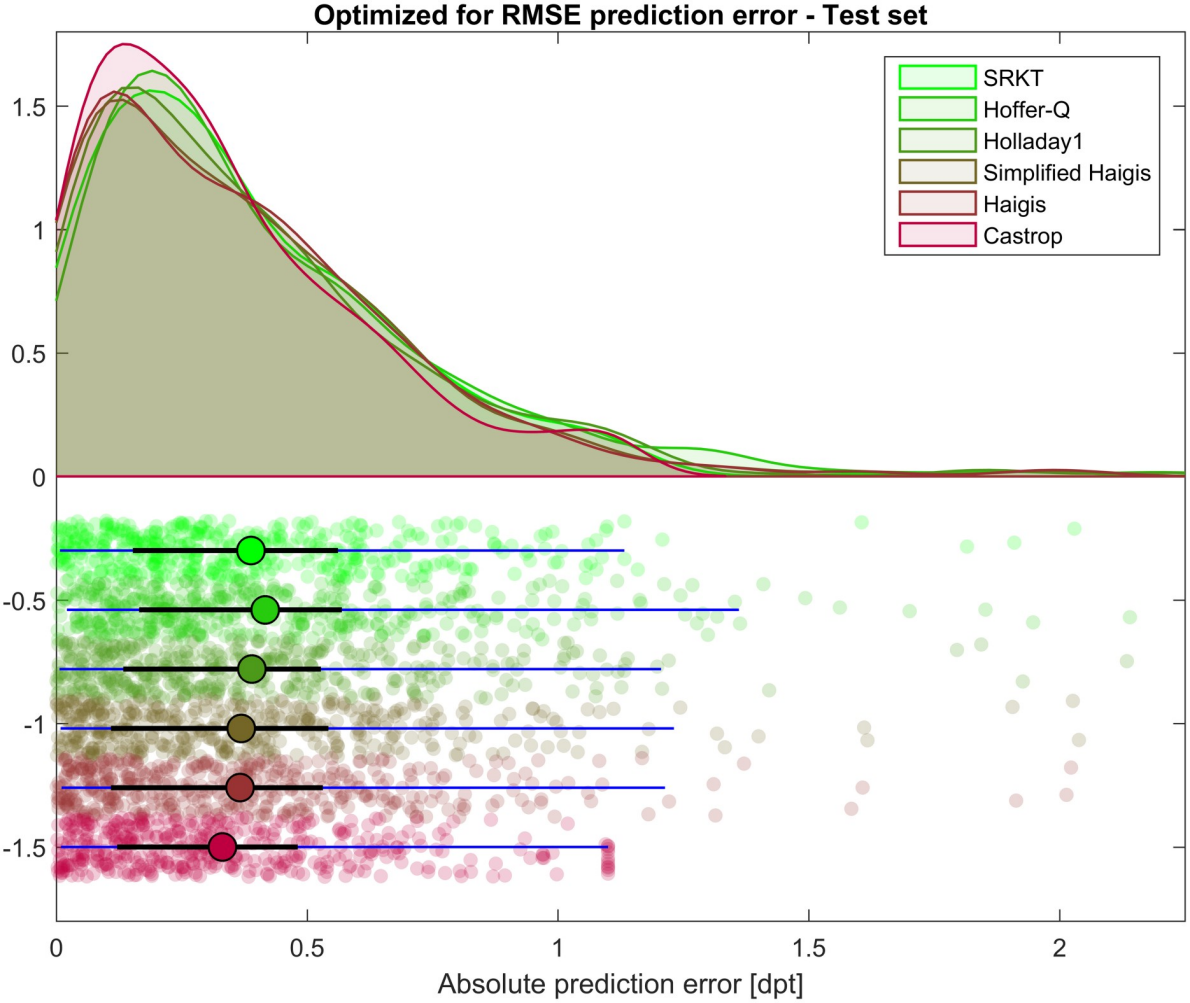
Absolute value of the prediction error absPE as the deviation of achieved spherical equivalent from the formula predicted refraction. Formula constants have been optimized on the training dataset (N = 1017) and cross-validated on the test set (N = 435). The upper part of the plot shows the approximated kernel density distribution for the 6 formulae under test, and the lower part the scatter of the absPE together with the median (circle), the quartiles (black line), and the 90% confidence interval (5% and 95% quantile, blue line). The blue lines in the lower plot representing the 90% confidence interval show that the absPE is largest with the Hoffer-Q formula and lowest with the Castrop and the SRKT formula.

Fig 2 displays the scatter of the absolute value of the formula prediction error absPE for the test data for all 6 formulae under test, together with the approximated kernel distribution, the median, the quartiles and the 90% confidence interval. Comparing absPE of the Castrop formula to absPE of the SRKT, Hoffer-Q, Holladay1, and Haigis formula with 1 and 3 optimized constants resulted in a p value of 0.0012, <0.001, <0.001, 0.004, and 0.008, respectively. Considering the Bonferroni correction for multiple testing yielded significant results for all comparisons.

**Fig 3:**
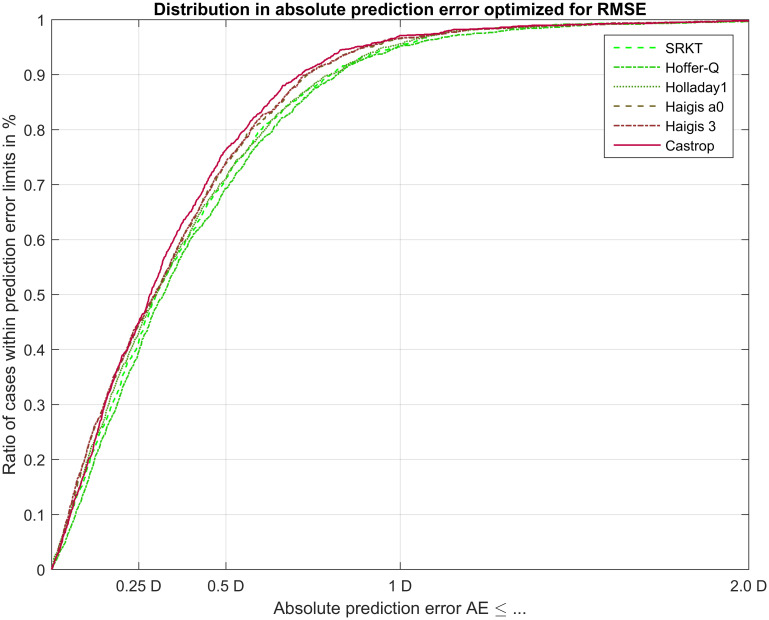
Performance of formula outcome in terms of absolute prediction error absPE. Formula constants have been optimized on the training dataset (N = 1017) and cross-validated on the test set (N = 435). The lines indicate the number of cases within the limits of absolute prediction error for the 6 different formulae under test, the closer the lines to 1 the more cases within limits. The ticks on the x axis indicate the typical thresholds of ±0.25, ±0.5, ±1.0 dpt as found in most publications. The plot indicates that between the limits of around 0.3 to 0.8 dpt of absolute prediction error the performance of the formulae under test show some differences, where the Castrop formula (solid line in red) appears to have a better performance compared to the Hoffer-Q formula (dashdotted line in dark green).

In Fig 3 the performance of formula outcome in terms of absolute prediction error absPE is shown for the N = 435 test data. The lines indicate the number of cases within the limits of absolute prediction error for the 6 different formulae under test, the closer the lines to 1 the more cases within limits. The ticks on the x axis indicate the typical thresholds of ±0.25, ±0.5, ±1.0 dpt as found in most publications.

**Fig 4:**
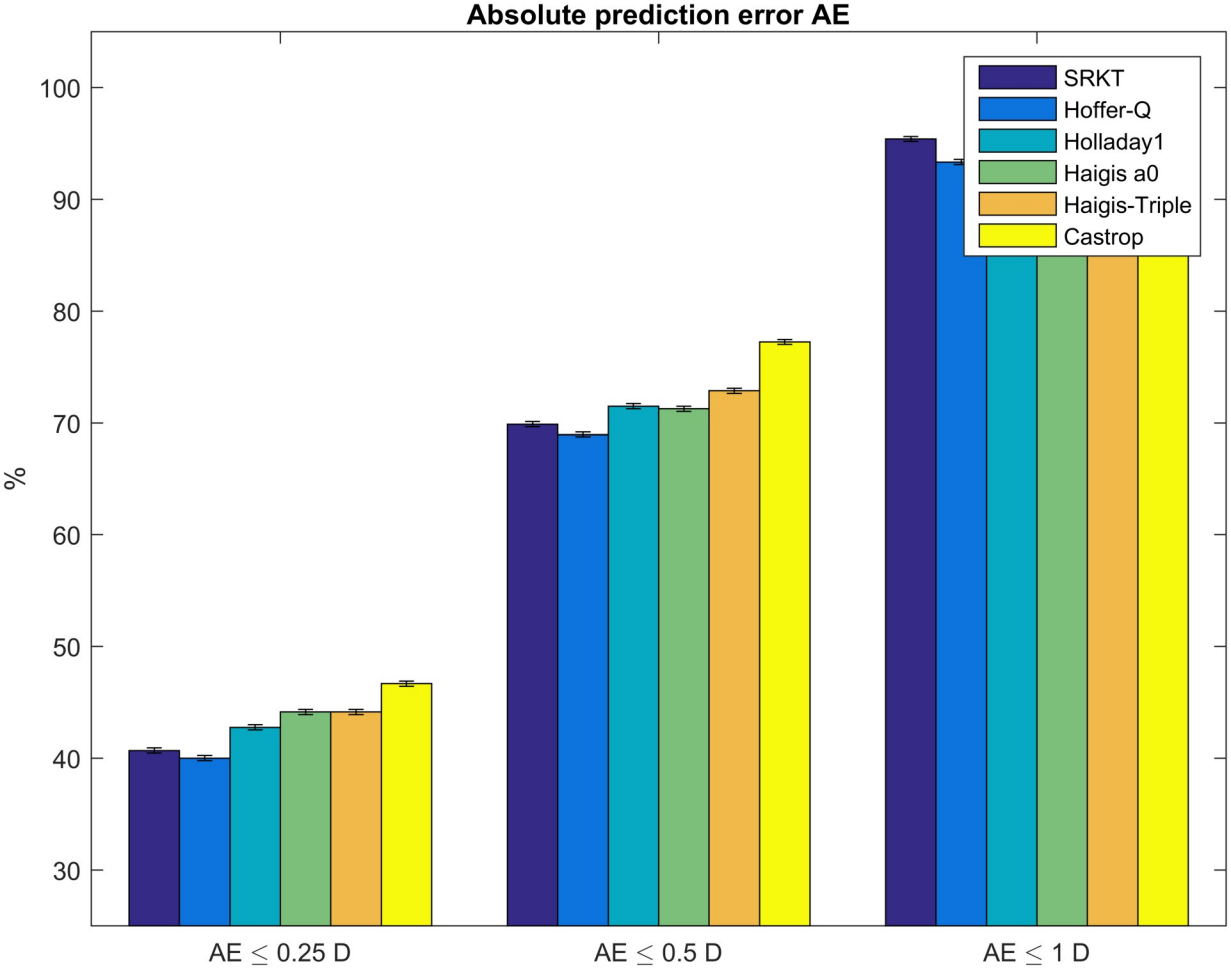
Ratio of cases within limits of absolute prediction error absPE for the 6 formulae under test. Formula constants have been optimized on the training set (N = 1017) and cross-validated on the test set (N = 435). The thresholds of ±0.25, ±0.5, ±1.0 dpt are used in accordance with the majority of publications on formula performance. For all 3 thresholds shown in this plot, the ratio of cases within limits shows a maximum difference of 5% between all 6 formulae.

In Fig 4 the ratio of cases with an absolute prediction error within limits of ≤0.25 dpt, ≤0.5 dpt, and ≤1.0 dpt is shown for the SRKT, the Hoffer-Q, the Holladay1, the simplified Haigis, the Haigis, and the Castrop formula. The bars correspond to the results of the test data, and the constants were optimized for the root mean squared prediction error rmsPE on the training data.

The formula performance indices FPI for the SRKT, Hoffer-Q, Holladay1, simplified Haigis, Haigis, and Castrop formula were 1.0588, 1.0157, 1.0624, 1,0898, 1,0952 and 1.1284, respectively.

## Discussion

Numerous formulae for calculation of intraocular lens power have been proposed in the last 20 years. In contrast to the basic formulae of Fyodorov [1] or Gernet [2] or the classical formulae of Sanders, Retzlaff and Kraff (SRKT), Hoffer (Hoffer-Q), Holladay (Holladay1) or Haigis (simplified Haigis with 1 optimized constant and Haigis formula with 3 optimized constants) [3–9], most of the formula authors nowadays do not disclose or publish the calculation strategy. At best they offer web based applications or software solutions for calculating the lenses. Such software tools do not allow batch calculations on a large set of patient data. Today, in many countries of the world classical formulae are increasingly being replaced by ‘modern’ calculation strategies such as the Barrett Universal II, Kane, Pearl, EVO, VRF or T2 formula [10, 11, 26]. To compare the prediction performance with other formulae it is necessary to enter the data manually, introducing a large risk of transcription errors. Additionally, a systematic optimization of constants is not possible for undisclosed formulae. Reading out the appropriate formula constant is mostly trial and error e.g. to eliminate the mean or median prediction error by varying the constants in the calculation scheme.

Withholding disclosure of formulae gives authors the option to adjust internal factors and offsets over the years in order to enhance the formula performance with more and more clinical data. This however bears the risk of different versions of the software yielding different outputs being available.

In the scientific world it is most appropriate to publish a new formula and to outline all options for adjusting the calculation. Such a publication includes the calculation strategy for the intraocular lens power, all the input parameters required for the calculations, and the adjustment parameters such as formula constants. Last but not least the formula author should advise how to optimize the formula constants based on a clinical dataset. But even in the classical formulae—with the exception of the SRKT formula—there is no guidance on how to optimize the formula constants. And in the original publication of the SRKT formula, the instruction is mostly based on a simplified back-calculation of the A constant similar to the empirical SRK2 formula.

In the present paper we describe the strategy of intraocular lens power calculation using the Castrop formula as described in Materials and Methods. The large dataset of 1452 eyes was split into training and test subsets [25]. The training data were used for formula constant optimization, and the test data for cross validation. In contrast, using the same dataset for constant optimization and validation risks ‘overfitting’ which may lead to an overestimation of an algorithm’s performance. Our results as presented in an explorative data analysis show that the Castrop formula yielded a slightly better performance compared to the classical SRKT, Hoffer-Q, Holladay1, or Haigis formula in terms of prediction error and absolute prediction error. In Fig 1 we see that the kernel distribution approximated to the prediction error shows the highest peak and a better concentration of the data with the Castrop formula. In contrast the Hoffer-Q formula seems to have a poorer performance. However, the distribution of all formulae is more or less symmetric about a prediction error of 0. In Table 2 the respective numbers are listed; in this table the mean describes any systematic offset in the formula predicted refraction and the standard deviation gives us a measure for the scatter of the individual values for the prediction error. With the absolute prediction error shown in Fig 2 the outcome is very similar: with the Castrop formula the entire distribution is closer to 0 which indicates a good performance compared to other formulae. The peak of the kernel distribution approximated to the absolute prediction error is larger and the distribution appears slimmer compared to the other formulae. In Table 3 the respective descriptive values are listed. As the distribution of the absolute prediction error values is strictly one-sided the mean and standard deviation are of minor interest. In contrast, the median value and the 95% confidence level are more relevant for interpretation of the results. From the data we find that 95% of all cases in the test data are within limits of 0.9 dpt of absolute prediction error. It is unfortunate that the Castrop formula cannot be compared directly with modern formulae as these formulae are not disclosed and therefore even with manual data entry in calculation software available online the standards for optimizing the formula constants are different.

### In conclusion

This study describes the calculation principle of the Castrop vergence formula for calculation of intraocular lens power based on biometric data of the eye. The concept is based on a pseudophakic model eye with 4 refractive surfaces: a refractive correction at spectacle plane, a meniscus lens for the cornea described with corneal front and back surface curvature and central thickness, and an intraocular lens implant. If tomographic data for the corneal back surface curvature and central corneal thickness are available and reliable, they can be directly used in the formula. Where they are not available or not reliable, the corneal back surface is derived from a fixed anterior to posterior corneal curvature ratio taken from the Liou-Brennan model eye. The Castrop formula uses 3 constants: the C constant in accordance with the Olsen formula, the Offset value H for a systematic shift of the IOL plane (which is mostly characterized by the optics and haptics design), and R for an offset in predicted refraction.

The Castrop formula together with 5 other classical lens power calculation formulae was applied to a large dataset of 1452 clinical measurements treated with one lens model. The dataset was split into a training set for constant optimization and a test set for cross-validation. Constant optimization for all formulae was performed using nonlinear optimization techniques aiming for a minimum root mean squared prediction error. In our explorative data analysis, the Castrop formula shows slightly superior results compared to the classical formulae in terms of prediction error and absolute prediction error. Further investigation of this calculation concept is needed with different datasets of different lenses to assess the advantages and limitations of this calculation concept.

## Supporting information

S1 Dataset (XLSX)

## References

[1] Fyodorov SN, Galin MA, Linksz A. Calculation of the optical power of intraocular lenses. Invest Ophthalmol 1975; 14: 625–628. 1150402

[2] Gernet H, Ostholt H, Werner H. Die präoperative Berechnung intraocularer Binkhorst-Linsen. 122. Vers. d. Ver. Rhein.-Westfäl. Augenärzte. Balve, Verlag Zimmermann 1970: pp. 54–55.

[3] Shammas HJ. Intraocular lens power calculations. Slack Inc 2004. ISBN-13: 978–1556426520.

[4] Sanders DR, Retzlaff JA, Kraff MC, Gimbel HV, Raanan MG. Comparison of the SRK/T formula and other theoretical and regression formulas. J Cataract Refract Surg 1990; 16(3):341–346. 10.1016/s0886-3350(13)80706-7 2355322

[5] Retzlaff JA, Sanders DR, Kraff MC. Development of the SRK/T intraocular lens implant power calculation formula. J Cataract Refract Surg 1990; 16(3):333–340. 10.1016/s0886-3350(13)80705-5 2355321

[6] Hoffer KJ. Steps for IOL power calculation. Am Intraocul Implant Soc 1980; 6(4):370. . 7440385

[7] Hoffer KJ. Intraocular lens calculation: the problem of the short eye. Ophthalmic Surg 1981; 12(4):269–272. . 7254770

[8] Hoffer KJ. The Hoffer Q formula: a comparison of theoretic and regression formulas. J Cataract Refract Surg 1993; 19(6):700–712. 10.1016/s0886-3350(13)80338-0 8271165

[9] Holladay JT, Prager TC, Chandler TY, Musgrove KH, Lewis JW, Ruiz RS. A three-part system for refining intraocular lens power calculations. J Cataract Refract Surg 1988; 14(1):17–24. 10.1016/s0886-3350(88)80059-2 3339543

[10] Savini G, Taroni L, Hoffer KJ. Recent developments in intraocular lens power calculation methods-update 2020. Ann Transl Med. 2020;8(22):1553. 10.21037/atm-20-2290 33313298 PMC7729321

[11] Wendelstein J, Hoffmann P, Hirnschall N, et al. Project hyperopic power prediction: accuracy of 13 different concepts for intraocular lens calculation in short eyes. Br J Ophthalmol 2021 27: bjophthalmol-2020-318272. 10.1136/bjophthalmol-2020-318272 33504489

[12] Vega Y, Gershoni A, Achiron A, Tuuminen R, Weinberger Y, Livny E, Nahum Y, Bahar I, Elbaz U. High agreement between Barrett Universal II calculations with and without Utilization of optional biometry parameters. J Clin Med. 2021;10(3):542. 10.3390/jcm10030542 33540639 PMC7867297

[13] Schröder S, Leydolt C, Menapace R, Eppig T, Langenbucher A. Determination of Personalized IOL-Constants for the Haigis Formula under consideration of measurement precision. PLoS One 2016; 11(7):e0158988. 10.1371/journal.pone.0158988 27391100 PMC4938522

[14] Aristodemou P, Knox Cartwright NE, Sparrow JM, Johnston RL. Intraocular lens formula constant optimization and partial coherence interferometry biometry: Refractive outcomes in 8108 eyes after cataract surgery. J Cataract Refract Surg 2011; 37(1):50–62. 10.1016/j.jcrs.2010.07.037 21183099

[15] Zhang JQ, Zou XY, Zheng DY, Chen WR, Sun A, Luo LX. Effect of lens constants optimization on the accuracy of intraocular lens power calculation formulas for highly myopic eyes. Int J Ophthalmol 2019; 12(6):943–948. 10.18240/ijo.2019.06.10 31236350 PMC6580219

[16] Hoffer KJ, Savini G. Update on Intraocular Lens Power Calculation Study Protocols: The Better Way to Design and Report Clinical Trials. Ophthalmology. 2020 9:S0161-6420(20)30638-2. 10.1016/j.ophtha.2020.07.005 32653457

[17] Liou HL, Brennan NA. Anatomically accurate, finite model eye for optical modeling. J Opt Soc Am A Opt Image Sci Vis 1997; 14(8):1684–1695. 10.1364/josaa.14.001684 9248060

[18] Norrby NE, Koranyi G. Prediction of intraocular lens power using the lens haptic plane concept. J Cataract Refract Surg 1997; 23(2):254–259. 10.1016/s0886-3350(97)80350-1 9113578

[19] Olsen T. Prediction of the effective postoperative (intraocular lens) anterior chamber depth. J Cataract Refract Surg 2006; 32(3):419–424. 10.1016/j.jcrs.2005.12.139 16631049

[20] Olsen T, Hoffmann P. C constant: new concept for ray tracing-assisted intraocular lens power calculation. J Cataract Refract Surg 2014; 40(5):764–773. 10.1016/j.jcrs.2013.10.037 24767910

[21] Levenberg K. A method for the solution of certain problems in least squares. Quart Appl Math 1944; 2:164–168.

[22] Marquardt D. An algorithm for least-squares estimation of nonlinear parameters. SIAM J Appl Math 1963; 11:431–441.

[23] Cooke DL, Cooke TL. Approximating sum-of-segments axial length from a traditional optical low-coherence reflectometry measurement. J Cataract Refract Surg 2019; 45(3):351–354. 10.1016/j.jcrs.2018.12.026 30851808

[24] Cooke DL, Cooke TL. A comparison of two methods to calculate axial length. J Cataract Refract Surg 2019 45(3):284–292. 10.1016/j.jcrs.2018.10.039 30851805

[25] Langenbucher A, Szentmáry N, Wendelstein J, Hoffmann P. Artificial Intelligence, Machine Learning and calculation of intraocular lens power. Klin Monbl Augenheilkd 2020; 23; 237(12):1430–1437. 10.1055/a-1298-8121 33231277

[26] Melles RB, Kane JX, Olsen T, Chang WJ. Update on intraocular lens calculation formulae. Ophthalmology 2019; 126(9):1334–1335. 10.1016/j.ophtha.2019.04.011 30980854

